# Contact tracing along with epidemiological and clinical characteristics of Novel corona virus 2019 in prisoners of Camp Jail, Lahore – A prospective case ascertaining study

**DOI:** 10.12669/pjms.38.3.4753

**Published:** 2022

**Authors:** Afshan Shahid, Shaza Shahid, Muhammad Ashraf, Khawwar Saeed

**Affiliations:** 1 Dr. Afshan Shahid, Department of Community Medicine, Services Institute of Medical Sciences (SIMS), Lahore, Pakistan; 2 Shaza Shahid, Avicenna Medical College, Lahore, Pakistan; 3 Dr. Muhammad Ashraf, Executive Director, Punjab Institute of Mental Health (PIMH), Lahore, Pakistan; 4 Dr. Khawwar Saeed, Consultant, Camp Jail. Lahore, Pakistan

**Keywords:** COVID-19, Contact tracing, Prisons, Secondary attack rate

## Abstract

**Background & Objectives:**

Prisons are reported as hub for communicable disease, as the closed environment, overcrowding, poor hygienic conditions facilitate the disease transmission. This study was conducted to describe contact tracing to identify, educate and manage COVID-19 in camp jail Lahore and to describe clinical and epidemiological features of disease in prisoners.

**Methods:**

After diagnosis of primary case of COVID-19 on 24^th^ March, 2020 in camp jail, 527 suspected cases were identified through contact tracing. The health department-initiated case identification through contact tracing, isolation of confirmed and suspected cases, and quarantine of exposed persons and establishment of 100 bedded hospital in jail for infection prevention and control and treatment. Baseline characteristics of primary case and secondary cases were described along with the secondary attack rate of infection.

**Results:**

Mean age of secondary cases was 36.9(11.5) years with mean stay of 14.9(13.6) months. Two third of the prisoners were from Punjab. 11 % were illiterate and almost half were under metric. 527 prisoners were labelled as suspected cases through contact tracing and 59 out of 527 suspected prisoners tested COVID positive through RT-PCR with few reporting mild respiratory symptoms. Fifty five out of 59 tested negatives on day-5 and all have uneventful recovery by day-21. Secondary attack rate was 11%.

**Conclusions:**

In order to prevent Covid-19 outbreaks, proactive containment and comprehensive contact tracing to identify monitor and manage cases and contacts, in incarcerated facilities like prisons is a public health solution to prevent and control large scale epidemic. Active monitoring for infected patients, and implementing timely infection prevention and control measures are mandatory for highly infectious Covid-19 in this vulnerable population.

## INTRODUCTION

Prisons are reported as hub for communicable disease, as the closed environment, overcrowding, poor hygienic conditions facilitate the disease transmission. Prisoners cannot only transmit disease amongst themselves but can be a source for the community too.^1^ Poor nutrition, mental stress, drugs and existing diseases make them vulnerable to infections.

The COVID -19 outbreak started from Wuhan, city of China in December 2019 and because of its communicability it was declared as pandemic by WHO in march 2020. Pakistan reported first case of COVID -19 on 27^th^ February 2020 and as on 7^th^ April 2020 total 4009 cases of COVID 19 are confirmed, out of which 2004 cases were from Punjab being the largest province of the country by population. About 500 cases of COVID -19 among prisoners have been reported during this pandemic from China and 70,000 prisoners were released from Iranian prisons to curtail the outbreak.^2^ Influenza outbreaks were reported since 1918 and usually starts by a newly inducted prisoner who is not isolated. Similar outbreaks have been reported from California^3^, Canada^4^, and Taiwan.^5^ Contact tracing followed by isolation, notification and treatment was reported as effective way for controlling previous epidemics of diseases like small pox^6^ and SARS.^7^

In Pakistan there are 89 prisons with 90,000 prisoners, 0ut 0f which 48,800 confined in jails of Punjab. So far, no study has been reported from any prison across Pakistan related to COVID-19 from this incarcerated population as studies have reported that these places were hub of many infectious diseases in Pakistan^8^ like hepatitis B and C, HIV^9^, syphilis, and Tuberculosis and termed as excellent venue for screening of existing infections and contact tracing strategy to curtail the spread of new infections. So far, no study has been published for COVID -19 in prisons. This study was conducted in Camp jail of Lahore which is being managed by services hospital Lahore for prevention, control and management of COVID -19 pandemic. This study aimed to describe the epidemiological features in terms of secondary attack rate of disease from the primary case and clinical features of confirmed cases of COVID 19 along with the contact tracing strategy undertaken to curtail the spread of the disease in prisoners.

## METHODS

### Study Design

This descriptive case ascertaining study^10^ was conducted in camp jail of Lahore housing 2970 cases at the time of study. A person with laboratory confirmation of COVID-19 infection, irrespective of clinical signs and symptoms was taken as confirmed case, which was further classified as a primary case (or index case). A contact who becomes a case with positive test result 24 hours or more after the latest positive test date of the primary case; or with onset of symptoms 24 hours or more after the latest onset date of the primary case. Study was conducted in district jail also known as camp jail located on main Firozpur Road, Lahore. At the time of study 2790 prisoners were housed in the jail with a capacity of 1000 prisoners designated by prisons department of Punjab. Primary case with flu like symptoms and suspicion of having COVID-19 was reported by jail medical officer to nearest Services hospital. The details of setting facility based 100 bedded isolation and treatment center are already published.^11^ Contact tracing was started 24^th^ March 2020 which was divided in four parts, identification, testing, notification, followed by follow up, monitoring and support. Demographic data, clinical signs and symptoms details, laboratory testing, and management data was collected on a questionnaire adopted from The World Health Organisations guidelines on responding to covid-19 in prisons.^11^,^12^ Data was collected by jail medical officer from the medical records on data collection sheet and patient were followed till the final outcome The suspected cases were tested through nasopharyngeal swab taken by a dedicated team of health department and testing was done in batches 31^st^ march to 4^th^ April 2020 due to limited capacity of the laboratory. Those testing positives were admitted in jail hospital established for COVID-19 treatment and test negative were quarantined in 192 single cells and vacant barracks for quarantine of two weeks. Blood complete picture, Liver and renal function tests as baseline were performed for infected prisoners within the jail laboratory. It was decided by the infectious disease specialist that chest X-ray and HRCT will only be performed for prisoners showing involvement of lower respiratory tract or fall in oxygen saturation levels.

Continuous data age, clinical tests measurements are presented as mean (SD) categorical variables like ethnicity, language, are presented as count (%). Secondary attack rates and symptomatic rates from all positives was calculated. Data was summarized using SPSS (version 23.0). Ethical Approval for the study was taken from IRB of Services Institute of Medical Sciences (IRB/2020/647/SIMS) ensuring full confidentiality of the data, and informed consent.

## RESULTS

Primary case was 33 years old male resident of Punjab, planning to travel to Italy was deported by Airport security forces on 8^th^ March 2020. He was rotated in different barracks as jail SOPs. the jail MO noticed flu like symptoms in him on 18^th^ March 2020, and was isolated by him. He notified this to jail authorities and health department with a suspicion of COVID-19. Joint planning was done between jail superintendent, home and health department. Services Hospital being the nearest hospital was assigned the duty to take up responsibility to manage further. Contact tracing was planned in order to curtail the disease outbreak. Demographic and clinical profile of cases is shown in Table-I.

**Table-I:** Baseline characteristics of COVID-19 positive prisoners at camp Jail (N=59).

Characteristics	Frequency (%age)	Normal range
***Age in years***, mean (SD)	36.9(11.5)	
Duration of imprisonment, mean (SD)	14.9(13.6)	
***Residence***		
Punjab	41 (69.49%)	
KPK	16(27.11%)	
Balochistan	1(1.70%)	
Azad Jammu Kashmir	1(1.70%)	
***Language***		
Pushto	30(50.84%)	
Punjabi	27(45.78%)	
Urdu	02(3.38%)	
***Education***		
Illiterate	11(18.64%)	
Primary	O7(11.29%)	
Metric	31(52.54%)	
Intermediate	04(6.77%)	
Bachelor	06(10.16%)	
***Symptoms***		
Fever	9(15.25%)	
Cough	6(10.1%)	
Runny Nose	6(10.1%)	
Sore throat	7(11.86%)	
Headache	4(6.77%)	
Lethargy	5(8.47%)	
***Co-morbidities***		
Diabetes	6(10.1%)	
Hypertension	4(6.77%)	
Cardiovascular disease	2(3.38%)	
Kidney Disease	1(1.69%)	
Blood disorder	1(1.69%)	
***Blood complete picture (BCP)***	Value, Mean(SD)	
Hemoglobin	14(1.38)	11.5-16g/dl
TLC	8.18(1.62)	4-11*10^^^9/l
Platelets	220(45.7)	150-400*10^^^9/l
Neutrophils	56.5(7.16)	40-75%
Lymphocytes	35.6(6.8)	20-50%
Monocytes	4.5(6.4)	02-10%
Eosinophils	1.17(.80)	1-6%
***Liver function tests***		
Serum Bilirubin	0.49(.21)	0.1-1.2mg/dl
ALT(SGPT)	28.7(18.2)	Less than 45mg/dl
AST(SGOT)	24.6(6.4)	Less than 35mg/dl
***Renal function tests***		
Serum Urea	24.6(6.4)	10-50mg/dl
Serum Creatinine	0.7(.30)	0.72-1.25mg/dl

### Risk Management

The team comprising of hospital administrator, infectious disease specialist and two consultants had a detailed meeting with jail superintendent. Shifting of almost 2000 non-contact prisoners was planned to reduce overcrowding and to have space available for quarantine of suspected contacts. Hundred-bedded hospital was planned in the jail for isolation of cases in barracks with most air ventilation and at the side of the jail. Infectious disease nurse gave training to house keeping staff for preparing disinfection solution and process of disinfection. Disinfection gate was installed at jail entrance with temperature monitoring of every visitor. Details of visitors were entered in a register at entry point which were only the hospital and jail staff as other visitors were banned. Detailed instructions were displayed in local language at different sites with hand washing facility. health awareness session of all the prisoners were taken to give them details about pandemic and different prevention and control measures to be followed.

### Contact tracing and identification

Contacts of the primary case were identified from all the barracks, and jail staff working there where he was rotated in last three days. Total 527 suspected cases were identified till 29^th^ march after one week of primary case diagnosis. As this covers the incubation period of Covid 19 so contact tracing was started among the prisoners who had a contact with the primary case. The family contacts of prisoners outside the prison were not investigated as there was a complete ban on prisoners to meet any outsider after the initial case was suspected to have Covid 19. Meanwhile the primary case was hospitalized for isolation. These suspected cases were quarantined and arrangements were made to have testing facility availability for this big number. As it was beginning of pandemic in Pakistan so resource constraints in terms of testing kits, trained staff and rapid testing was not available. The testing process started on 31st March and completed on 4^th^ April identifying 59 confirmed cases of COVID -19. These cases were isolated in newly established jail hospital with attached donning and doffing area and designated point for infectious waste storage. Waste was collected twice by the waste disposal team of services hospital for disposal with separate entry and exit point.

### Notification and management

All cases were notified to health department and jail authorities. Base line investigations were done and oxygen saturations was checked thrice daily. Main symptoms reported were flu, sore throat, fever and headache which all settled with antipyretics. Cap Azomax 500mg daily with antimalarial drugs were administered as treatment options available through national guidelines. Meals were provided by a local philanthropic organization three times a day in disposable containers.

### Follow up

All prisoners were very happy as hospital beds with mattresses, so much care and healthy meals all times was unexpected treatment by them. PCR was repeated at day five and 55 out of 59 turned out to be negative and four remained positive. These four four positive cases were further isolated in a separate barrack and remaining negative cases were still confined in hospital so that they can be retested on day 14 to declare them COVID-19 free. Meanwhile all those who tested negative out of 527 were also under observation and none of them developed any symptoms. None out of 59 prisoners had any lower respiratory symptoms so no X rays or HRCTs were done. All 59 cases were tested on day 14 and finally on 2^nd^ May 2020 all were declared Covid free.

Home department transferred few cases from other jails of PUNJAB but video presentation was given by Dr. Mehmood Ayyaz to all Jail superintendents to manage COVID cases in existing facilities to avoid cross infections.

### Secondary Attack Rate

Secondary attack rate was 11.19 % calculated by excluding the primary case from the numerator and denominator also.

## DISCUSSION

Our study reported successful control of Covid 19 by timely contact tracing for early identification, isolation and treatment of the cases who developed infection after being exposed to a single prisoner, and resulted in mild infection due to early management. Early identification of primary case and isolation also prevented the spread. Earlier studies from previous epidemics in past have also shown that the infection in prison is mostly introduced from a new prisoner.^3^ Contact tracing and timely collaboration between health department and prison department was a leading factor for establishing in prison treatment facility which averted future contact with the primary case and was a reason that a significant proportion of contacts remained disease free.

Two third of the prisoners were under matric, and were incarcerated for more than a year, which makes them more vulnerable to respiratory diseases and other common cold and influenza type infections. Stay in such deplorable conditions and factors like overcrowding, poor ventilation which makes the spread common is well recognized in studies conducted in prisons.^13^,^14^ Significant proportion (> 85%) didn’t develop the infection which may be attributed to their natural immunity or resistance to infection because of past exposures. Early isolation of the primary case has reduced the disease transmission is comparable with other studies reporting active monitoring and contact tracing from Ghana^15^ and USA.^16^ The symptoms suggest that our patients had mild symptoms like fever, sore throat, runny nose and headache and that too among less than one third of the isolated cases. Increasing evidence is suggestive of similar findings as that mild clinical symptom could be more frequent in cases of COVID-19 as compared to SARS- CoV and MERS-CoV.^16^

Initial planning to shift non-contact prisoners to other facilities was a good initiative to reduce overcrowding in the jail and to reduce the risk of further infection transmission also helped to limit the spread although Iran one of our neighboring country even released 70,000 prisoners to avoid in house transmission.^17^ Our experience of working in an incarcerated facility was operationalized at two levels, firstly Contact tracing along with proper quarantine of the suspected individual, clinical monitoring of those who tested negative during their quarantine period along social distancing among the jail staff including restricted resident movements, and no visitors was very effective. Secondly Health awareness sessions of prisoners regarding pandemic as they do not have access to information, along with infection prevention and control training of housekeeping staff. Ensuring PPE use during their duty, designating proper donning and doffing area with separate storage, entry and exit point for infectious waste disposal further enhanced the efficacy of the plan implemented for infection control which was followed according to the WHO guidelines issued for the management of COVID -19 in prisons.^10^ The impact of these policies in protecting long- term care facilities could only be ensured through strict monitoring and continuous reinforcement by retraining’s and repeated health awareness sessions Nonpharmaceutical interventions implemented at the start of any epidemic are most effective strategies in previous epidemics.

Secondary attack rate (SAR) which measures the spread of infection in a closed place like households or detention facilities was found to 11.6% in our study which is close to the SAR calculated to be 16.3% in adult households in China.^18^ SAR of 13% was reported from USA for Influenza A in 2009.^19^ We conducted RT-PCR on day 5, day 14 and day 21 to declare the cases disease free. This follow up was only possible in prison otherwise it is difficult to follow cases in countries like Pakistan in population-based studies.

This study provides valuable steps taken which can be followed in other prisons to avert the infection across Pakistan. This opportunity to work with Prisons being a walled place provide excellent venue for screening of many other infectious diseases like T.B, hepatitis and HIV etc to break the link of disease transmission to community by those prisoners released after a short stay and amongst the other prisoners if they have a long stay. They can also infect other prisons if transferred to other jails. The happiness and comfort enjoyed being in the hospital with hospital bed and mattress, round the clock care and good food portrays the poor living conditions being faced by them which is against basic human rights. Over 10 million people are incarcerated worldwide. The UN Basic Principles for the Treatment of Prisoners states that prisoners “shall have access to the health services available in the country without discrimination on the grounds of their legal situation.”^20^Alarming living conditions prison populations reveal that healthcare services are haggard.

### Limitations of the study

It was identified as a single study conducted in only one jail of Lahore although similar measures should have been taken at least in the Kot Lakhpat jail of Lahore. Few delays were encountered like primary case diagnosed on 24^th^ March but testing started for the suspected prisoners almost a week later because of lack of testing facilities as these were the initial days of pandemic in Pakistan and having few overburdened designated testing facilities. If all suspected could have been tested simultaneously, along with the screening of their contacts outside the prison prior to diagnosis of the primary case would have given a much better picture of secondary cases that either they directly got infection from the primary case, or any outside contact or in our scenario may have contracted infection from a secondary case during the incubation period who may be negative initially but got infected being quarantined in the same barrack. The total number is presented by total positives in all reports during a week time, so the timing of disease onset in the facility cannot be specified.

## CONCLUSION

In order to prevent Covid-19 outbreaks, proactive measures of contact tracing, early testing, followed by isolation, quarantine, treatment and follow up in incarcerated facilities is a public health solution to prevent and control large scale epidemic. Active monitoring for infected patients, and implementing timely infection prevention and control measures within the prison are mandatory for highly infectious Covid-19 in this vulnerable population.

### Authors’ Contribution:

**AS:** Designed, statistical analysis, editing of manuscript as per scientific guidelines, final approval and accountable for integrity of this work.

**SS, KS, MA:** Data collection, Data entry, writing first draft of manuscript.
